# Supplementary material for: A broadly neutralizing humanized ACE2-targeting antibody against SARS-CoV-2 variants
Source: Nat Commun. 2021 Aug 17;12:5000. doi: 10.1038/s41467-021-25331-x (PMC8371079; doi:10.1038/s41467-021-25331-x)
Supplement: Supplementary file 1 — Supplementary information [file 41467_2021_25331_MOESM1_ESM.pdf]

Supplementary information

A broadly neutralizing humanized ACE2-targeting antibody against SARS-CoV-2 variants

Yanyun Du[1,10], Rui Shi[2,10], Ying Zhang[3,10], Xiaomin Duan[2,4], Li Li[5], Jing Zhang[5], Fengze Wang[2,4], Ruixue Zhang[3], Hao Shen[3], Yue Wang[2,4], Zheng Wu[2,6], Qianwen Peng[1], Ting Pan[1], Wanwei Sun[1], Weijin Huang[7], Yue Feng[8], Hui Feng[5], Junyu Xiao[3], Wenjie Tan[9]*, Youchun Wang[7]*, Chenhui Wang[1]*, and Jinghua Yan[2,4]*

[1]Key Laboratory of Molecular Biophysics of the Ministry of Education, National Engineering Research Center for Nanomedicine, College of Life Science and Technology, Huazhong University of Science and Technology, Wuhan 430074, China.

[2]CAS Key Laboratory of Microbial Physiological and Metabolic Engineering, Institute of Microbiology, Chinese Academy of Sciences, Beijing 100101, China.

[3]State Key Laboratory of Protein and Plant Gene Research, School of Life Sciences, Peking-Tsinghua Center for Life Sciences, Beijing Advanced Innovation Center for Genomics, Peking University, Beijing 100871, China.

[4]University of Chinese Academy of Sciences, Beijing 100049, China.

[5]Shanghai Junshi Biosciences Co. Ltd, Shanghai 200126, China.

[6]Institute of Physical Science and Information, Anhui University, Hefei, 230039, China.

[7]Division of HIV/AIDS and Sex-transmitted Virus Vaccines, Institute for Biological

Product Control, National Institutes for Food and Drug Control (NIFDC) and WHO Collaborating Center for Standardization and Evaluation of Biologicals, Beijing 102629, China.

[8]Beijing Advanced Innovation Center for Soft Matter Science and Engineering, Beijing Key Laboratory of Bioprocess, State Key Laboratory of Chemical Resource Engineering, College of Life Science and Technology, Beijing University of Chemical Technology, Beijing 100029, China.

[9]NHC Key Laboratory of Biosafety, National Institute for Viral Disease Control and Prevention, Chinese Center for Disease Control and Prevention, Beijing 102206, China.

[10]These authors contributed equally: Yanyun Du, Rui Shi, Ying Zhang.

*These authors jointly supervised this work: tanwj@ivdc.chinacdc.cn (W.T.), wangyc@nifdc.org.cn (Y.W.), wangchenhui@hust.edu.cn (C.W.) and yanjh@im.ac.cn (J.Y.)

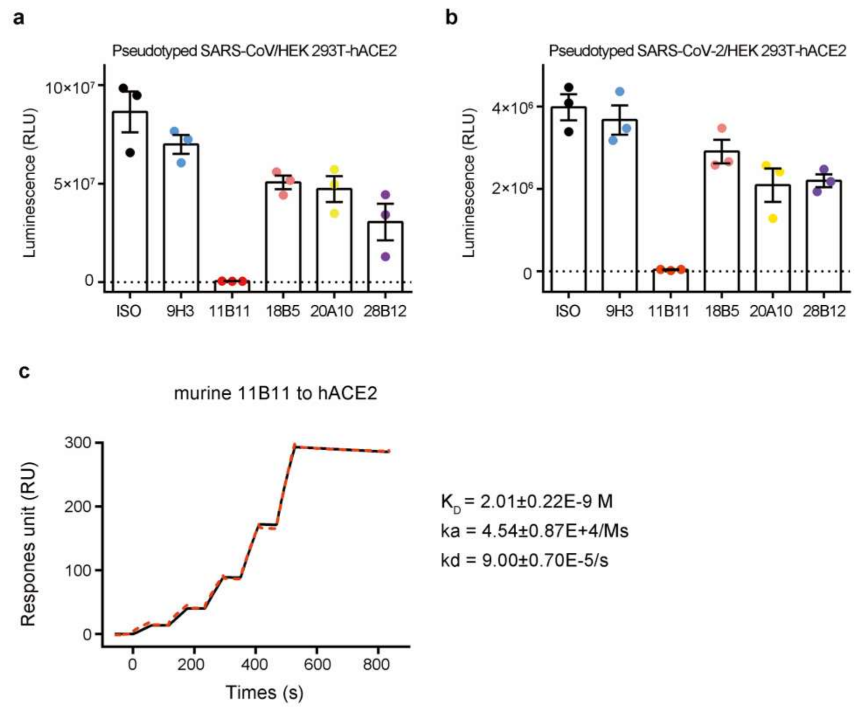

Supplementary Fig. 1 Screening of hACE2-blocking hybridoma supernatants. a-b, HEK293T-hACE2 were incubated with hybridoma or control supernatants. SARS-CoV and SARS-CoV-2, pseudovirus were then added to relevant cells. After incubation, inhibition potencies of MAb were evaluated in a luciferase reporter assay. **c,** The binding kinetics of murine 11B11 to hACE2 was assessed using a single-cycle model. MAb was captured on the chip while serial dilutions of hACE2 proteins then flowed over the chip surface. The kinetic parameters were labeled accordingly. Mean values of three biological replicates±SEM (standard error of the mean) are shown for a and **b**. Data are representative of three independent experiments.

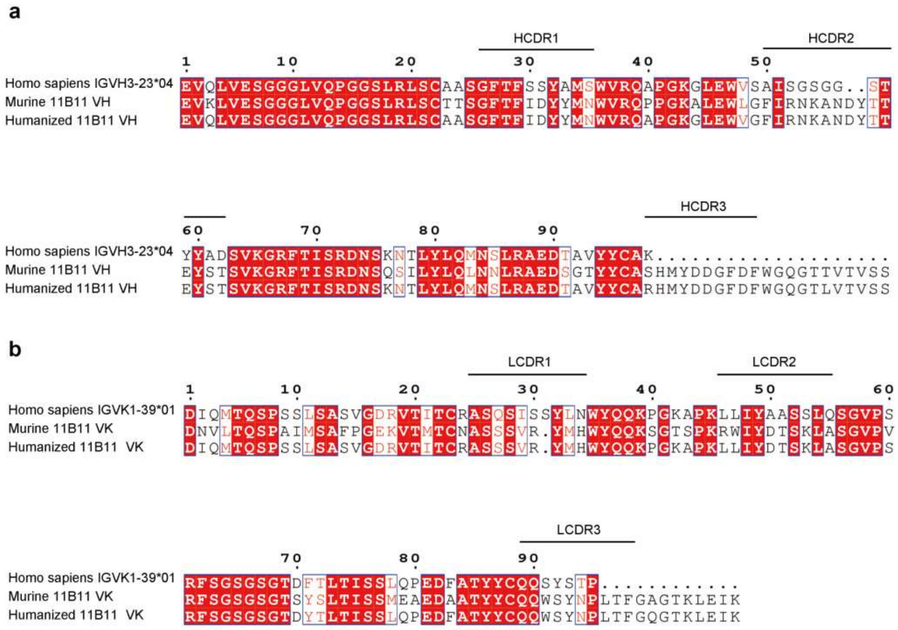

Supplementary Fig. 2 Humanization strategy of MAb 11B11. a-b, Sequence alignments highlighting the humanization strategy of murine 11B11 by retaining all the CDRs and substituting the remaining amino acids with the corresponding residues of the human immunoglobulins. The human IGHV2-23*04, which exhibits the high sequence identity to murine 11B11 in heavy chain, was selected as the humanization backbone for the H chain, while IGKV1-39*01 was selected as the humanization backbone for the L chain.

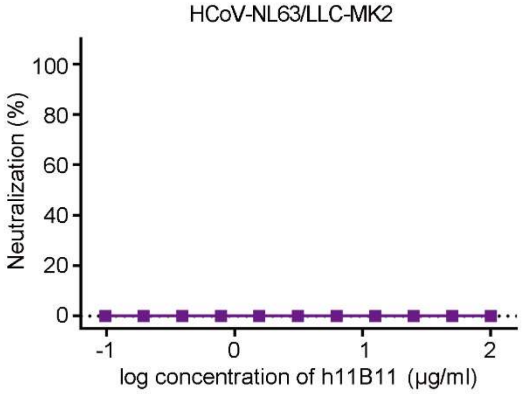

Supplementary Fig. 3 h11B11 exhibits no neutralization to HCoV-NL63 in vitro. LLC-MK2 cells were incubated with serially diluted h11B11 protein. 20 TCID50 of HCoV-NL63 in 50 μl were added in the mixtures. The CPE was observed in all samples after three days of incubation.

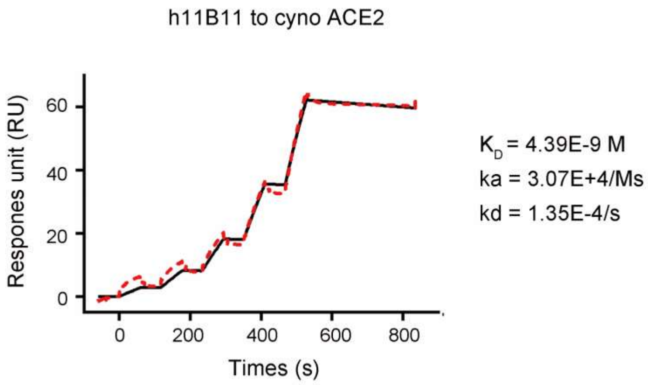

Supplementary Fig. 4 Binding affinity of h11b11 to cynomolgus ACE2. MAb was captured on the chip while serial dilutions of cyno ACE2 proteins then flowed over the chip surface. The kinetic parameters were labeled accordingly.

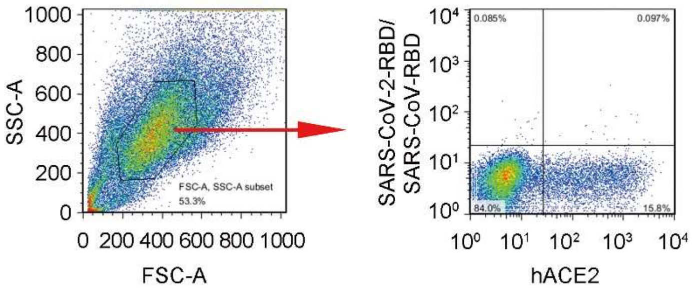

Supplementary Fig. 5 Gating strategy of flow cytometry to assay the blocking functions. HEK293T-ACE2 cells were stained and analyzed by flow cytometry. The cell was progressively gated to identify single cells and hACE2+ or SARS-CoV-2-RBD/SARS-CoV-2-RBD+ cells as shown in the right panel.

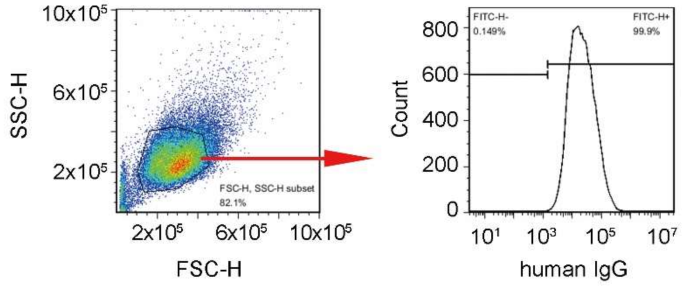

Supplementary Fig. 6 Gating strategy of low cytometry to detect the hACE2 level on the cell surface. HEK293T-ACE2 cells were stained and analyzed by flow cytometry. The cell was progressively gated to identify single cells and Alexa Fluor^{TM} 488-h11B11+ cells as shown in the right panel.

Supplementary Table 1 Measurement of enzymatic kinetic constants of hACE2 in the presence of MAbs and inhibitor.

hACE2	(ng)	40	40	40	40	40	40
h11B11	(μg/ml)	0	100	200	400	0	0
Isotype IgG	(μg/ml)	0	0	0	0	400	0
MLN-4760	(μM)	0	0	0	0	0	10
Vmax	($\mu M \cdot min^{-1}$)	0.77±0.11	1.52±0.04	1.47±0.07	1.46±0.06	1.45±0.05	n.d
Km	(μM)	38.99±5.62	45.18±1.27	42.12±1.81	41.58±2.00	41.30±0.78	n.d
Kcat	(S^{-1})	2.71±0.41	5.40±0.15	5.21±0.24	5.18±0.02	5.15±0.16	n.d
Kcat/Km	($\mu M^{-1} \cdot S^{-1}$)	0.07±0.00	0.12±0.00	0.12±0.00	0.13±0.00	0.13±0.00	n.d

Parameters shown for each condition represent the average of three replicates±SEM (standard error of the mean). n.d means the values are not detectable due to poor binding ability.

Supplementary Table 2 Data collection and refinement statistics.

	ACE2/h11B11-Fab
**Data collection**	
Space group	P 2_1 2_1 2_1
Cell dimensions	
a, *b*, *c* (Å)	98.779, 115.462, 224.668
Resolution (Å)	3.8*
R_{merge}	0.415 (1.739)
R_{pim}	0.119 (0.493)
$I / \sigma I$	5.5 (1.3)
Completeness (%)	100.0 (100.0)
Multiplicity	13.08 (13.4)
Wilson B-factor	95.0
**Refinement**	
Reflections used in refinement	25788 (2435)
Reflections used for R_{free}	1284 (122)
R_{work} / R_{free}	0.276 / 0.327
No. of non-hydrogen atoms	
Protein	16055
Metals	2
Solvent	0
Protein residues	2016
B-factors	
Protein	118.68
Ligands	148.40
Number of TLS groups	1
R.m.s deviations	
Bond lengths (Å)	0.003
Bond angles (°)	0.69
Ramachandran	
Favored (%)	97.1
Allowed (%)	2.8
Outliers (%)	0.1

Diffraction data from one crystal was used for structure determination.

*Values in parentheses are for the highest-resolution shell.
