# Supplementary material for: A broadly neutralizing humanized ACE2-targeting antibody against SARS-CoV-2 variants
Source: Nat Commun. 2021 Aug 17;12:5000. doi: 10.1038/s41467-021-25331-x (PMC8371079; doi:10.1038/s41467-021-25331-x)
Supplement: Supplementary file 3 — Reporting Summary [file 41467_2021_25331_MOESM3_ESM.pdf]

nature research

Corresponding author(s): Prof. Jinghua Yan

Last updated by author(s): Jul 16, 2021

Reporting Summary

Nature Research wishes to improve the reproducibility of the work that we publish. This form provides structure for consistency and transparency in reporting. For further information on Nature Research policies, see our Editorial Policies and the Editorial Policy Checklist.

Statistics

For all statistical analyses, confirm that the following items are present in the figure legend, table legend, main text, or Methods section.

n/a	Confirmed	
[ ]	[x]	The exact sample size (*n*) for each experimental group/condition, given as a discrete number and unit of measurement
[ ]	[x]	A statement on whether measurements were taken from distinct samples or whether the same sample was measured repeatedly
[ ]	[x]	The statistical test(s) used AND whether they are one- or two-sided<br>*Only common tests should be described solely by name; describe more complex techniques in the Methods section.*
[x]	[ ]	A description of all covariates tested
[x]	[ ]	A description of any assumptions or corrections, such as tests of normality and adjustment for multiple comparisons
[ ]	[x]	A full description of the statistical parameters including central tendency (e.g. means) or other basic estimates (e.g. regression coefficient) AND variation (e.g. standard deviation) or associated estimates of uncertainty (e.g. confidence intervals)
[ ]	[x]	For null hypothesis testing, the test statistic (e.g. *F*, *t*, *r*) with confidence intervals, effect sizes, degrees of freedom and *P* value noted<br>*Give P values as exact values whenever suitable.*
[x]	[ ]	For Bayesian analysis, information on the choice of priors and Markov chain Monte Carlo settings
[x]	[ ]	For hierarchical and complex designs, identification of the appropriate level for tests and full reporting of outcomes
[x]	[ ]	Estimates of effect sizes (e.g. Cohen's *d*, Pearson's *r*), indicating how they were calculated

Our web collection on statistics for biologists contains articles on many of the points above.

Software and code

Policy information about availability of computer code

Data collection	Biacore 8K Control Software 2.0.15.12933, BD FACSDiva Software v8.0.3, CLARIOstar Plus 5.61, Beckman CytExpert 2.3.0.84, GLoMax 1.9.3, ecgAUTO v3.3.0.20, and XDS Program Package (Jan 31,2020) were used for data collection.
Data analysis	QuanStudio Design&Analysis Software v1.5.1, GraphPad Prism Version 6.0, Biacore Insight Evaluation 1.0.5.11069, HKL2000 v715, Phyre2 (www.sbg. bio.ic.ac.uk/phyre2), Coot 0.8.9, Phenix 1.10.1-2155, PyMOL 2.3.3, and FlowJo 7.6.1 were used for data analysis.

For manuscripts utilizing custom algorithms or software that are central to the research but not yet described in published literature, software must be made available to editors and reviewers. We strongly encourage code deposition in a community repository (e.g. GitHub). See the Nature Research guidelines for submitting code & software for further information.

Data

Policy information about availability of data

All manuscripts must include a data availability statement. This statement should provide the following information, where applicable:

- Accession codes, unique identifiers, or web links for publicly available datasets
- A list of figures that have associated raw data
- A description of any restrictions on data availability

Source data are provided with this paper. The authors declare that the data supporting the findings of this study are available within this paper or are available from the corresponding author (Jinghua Yan, yanjh@im.ac.cn and Chenhui Wang, wangchenhui@hust.edu.cn) upon reasonable request. The accession number for the atomic coordinates and diffraction data reported in this study is PDB 7E7E.The sequences of h11B11 have been deposited in GenBank with the accession codes MZ514137 and MZ514138 are provided with this paper. All the codes are releasing.

Field-specific reporting

Please select the one below that is the best fit for your research. If you are not sure, read the appropriate sections before making your selection.

☒ Life sciences ☐ Behavioural & social sciences ☐ Ecological, evolutionary & environmental sciences

For a reference copy of the document with all sections, see nature.com/documents/nr-reporting-summary-flat.pdf

Life sciences study design

All studies must disclose on these points even when the disclosure is negative.

Sample size	Sample sizes were estimated from other experiments obtained by similar experiments performed in former publications of our group. (Citations listed below)<br>In vitro studies: Sample sizes were determined based on pilot studies results and on previous similar studies from our lab.<br>In vivo studies: Animal experiments were conducted by using 8 mice and 2 cynomolgus monkeys per group. This sample sizes in previously and similar studies, have given statistical significant results. For ethical reasons, the minimum number of animals necessary to achieve the scientific objectives was used.<br>Citations:<br>1. doi: 10.1038/s41467-021-21037-2<br>2. doi: 10.1016/j.cell.2020.06.035<br>3. doi: 10.1038/s41586-020-2381-y<br>4. doi: 10.1038/s41564-019-0411-z
Data exclusions	No data were excluded.
Replication	All experiments were performed with sufficient biological or technical replications in order to demonstrate statistical significance. Number of replicates in each experiment is indicated in the corresponding figure legends.<br>All experiments were successfully replicated.
Randomization	The hACE2 transgenic mice were randomly divided into five groups. Cynomolgus monkeys were divided into two groups according to body weight. For the in vitro experiments other than animal studies, randomization is not necessary.
Blinding	The investigators were blinded to the study.

Reporting for specific materials, systems and methods

We require information from authors about some types of materials, experimental systems and methods used in many studies. Here, indicate whether each material, system or method listed is relevant to your study. If you are not sure if a list item applies to your research, read the appropriate section before selecting a response.

Materials & experimental systems

n/a	Involved in the study	
☐	☒	Antibodies
☐	☒	Eukaryotic cell lines
☒	☐	Palaeontology and archaeology
☐	☒	Animals and other organisms
☒	☐	Human research participants
☒	☐	Clinical data
☒	☐	Dual use research of concern

Methods

n/a	Involved in the study	
☒	☐	ChIP-seq
☐	☒	Flow cytometry
☒	☐	MRI-based neuroimaging

Antibodies

Antibodies used	anti-His-APC: Miltenyi Biotec, Cat No: 130-119-782, clone:GG11-8F3.5.1, Lot No:5200407730; goat anti-human IgG (H+L)-Alexa Fluor 488: Invitrogen, Cat No: A11013, Lot No:1885920; rabbit anti-ACE2 pAb: Proteintech, Cat No: 21115-1-AP, Lot No:00096425; rabbit anti-Na/K ATPase : Proteintech, Cat No: 14418-1-AP, Lot No:00083541; Anti-α-Tubulin: CST, Cat No: 2125, clone:11H10.
Validation	All antibodies are commercially available and were commercially validated.<br>Anti-His-APC (Miltenyi Biotec, Cat No: 130-119-782, clone:GG11-8F3.5.1):<br>Validation: PMID: 33556148 (FC)<br><br>Goat anti-human IgG (H+L)-Alexa Fluor 488: Invitrogen, Cat No: A11013<br>Validation: PMID: 28242217 (FC)

Anti-α-Tubulin: CST, Cat No: 2125
Validation: https://www.cellsignal.cn/products/primary-antibodies/a-tubulin-11h10-rabbit-mab/2125?site-search-type=Products&N=4294956287&Ntt=2125&fromPage=plp&_requestid=3237937

Rabbit anti-Na/K ATPase : Proteintech, Cat No: 14418-1-AP
Validation: PMID: 32574956 (western blot)

Rabbit anti-ACE2 pAb: Proteintech, Cat No: 21115-1-AP
Validation: PMID: 32966801 (western blot)

Eukaryotic cell lines

Policy information about cell lines

Cell line source(s)	HEK293T (CRL-3216), Vero E6 (CRL-1586), and LLC-MK2 (CRL-7) were obtained from ATCC. Sf21 (B82101)and Hi5(B85502) insect cells were purchased from Invitrogen. HEK293T-ACE2 was bought from Sino Biological (OEC001).
Authentication	Cell lines were authenticated by short tandem repeat profiling.
Mycoplasma contamination	All cell lines are negative for mycoplasma.
Commonly misidentified lines (See ICLAC register)	The cell lines used in this study do not appear on the ICLAC register.

Animals and other organisms

Policy information about studies involving animals; ARRIVE guidelines recommended for reporting animal research

Laboratory animals	40 hACE2 transgenic mice (female, 30-week-old) were housed in BSL-3 lab, the ambient temperature is between 18-25°C, the humidity is between 40-80%, and the environmental light/dark cycle is 12h light, 12h dark. Four cynomolgus monkeys (male, three-year-old) were housed in the non-GLP laboratory.
Wild animals	The study does not involve wild animals.
Field-collected samples	No field-collected samples were used in this study.
Ethics oversight	The mice were used according to protocols approved by the Institutional Animal Care and Use Committee of Chinese Academy of Sciences. The cynomolgus monkeys were used according to protocols approved by Jiangsu Tripod Preclinical Research Laboratories Co., LTD.

Note that full information on the approval of the study protocol must also be provided in the manuscript.

Flow Cytometry

Plots

Confirm that:

- [x] The axis labels state the marker and fluorochrome used (e.g. CD4-FITC).
- [x] The axis scales are clearly visible. Include numbers along axes only for bottom left plot of group (a 'group' is an analysis of identical markers).
- [x] All plots are contour plots with outliers or pseudocolor plots.
- [x] A numerical value for number of cells or percentage (with statistics) is provided.

Methodology

Sample preparation	To test the activity of antibodies to block the binding between ACE2 and SARS-CoV-RBD, or SARS-CoV-2-RBD. HEK293T cells were transiently transfected with pEGFP-N1-ACE2 plasmids. After 24 hours, cells were collected and incubated with 10 μg/ml h11B11 protein or isotype IgG at 37ºC for 30 min, followed by incubation with 200 ng/ml RBD proteins at 37ºC for another 30 min. After washing three times, the cells were incubated with APC-conjugated anti-His antibody （1:200, Miltenyi Biotec) for another 30 min. Then, the cells were analyzed using flow cytometry (BD FACS Canto™ II).<br>To test whether the h11B11 antibody has any impact on the cell-surface expression of hACE2, HEK293T-hACE2 cells were incubated with different concentrations (10 μg/ml or with five-fold serial dilutions ranging from 10 μg/ml to 0.64 ng/ml) of h11B11 at 37ºC in DMEM with 10% FBS for 4 h or 24 h. Then the cells were washed with FACS buffer (PBS, 1% BSA, 2 mM EDTA) and incubated with 10 μg/ml h11B11 antibody or isotype IgG at 4ºC for 60 min. After washing three times, cells were incubated with Alexa FluorTM488 goat anti-human IgG (H+L) antibody (1:200, Invitrogen) at 4ºC for another 30 min. Then, the cells were washed twice and resuspended in 200 μl FACS buffer for flow cytometry analysis (Beckman CytoFLEX S).
Instrument	Beckman CytoFLEX S and BD FACSCanto™ II
Software	FlowJo 7.6.1

Cell population abundance	We analyzed about 40,000 stained cells of each sample.
Gating strategy	For blocking assays, SARS-CoV-RBD+ or SARS-CoV-2-RBD+ HEK293T-hACE2 cells were gated as ACE2-GFP+ and RBD-APC+. More Information available on Methods section. For detecting the hACE2 level on the cell surface, HEK293T-ACE2 cells were stained and analyzed by flow cytometry. The cell was progressively gated to identify single cells and Alexa FluorTM 488-h11B11+ cells as shown in the right panel.

☒ Tick this box to confirm that a figure exemplifying the gating strategy is provided in the Supplementary Information.
